# An explorative analysis of pharmacovigilance data of oxytocin and its analogue carbetocin, with a focus on haemodynamic adverse effects

**DOI:** 10.1007/s11096-023-01587-9

**Published:** 2023-05-10

**Authors:** Dominik Stämpfli, Rebecca Dommrich, Sharon Orbach-Zinger, Andrea M. Burden, Michael Heesen

**Affiliations:** 1 Pharmacoepidemiology, Institute of Pharmaceutical Sciences, Department of Chemistry and Applied Biosciences, ETH Zurich, Vladimir-Prelog-Weg 4, 8093 Zürich, Switzerland; 2 Hospital Pharmacy, Kantonsspital Baden, Baden, Switzerland; 3 Department of Anesthesia, Beilinson Hospital, Rabin Medical Center Associated With Sakler Medical School, Tel Aviv University, Tel Aviv, Israel; 4 Department of Anaesthesia, Kantonsspital Baden, Baden, Switzerland

**Keywords:** Carbetocin, Hemodynamics, Obstetrics, Oxytocin, Pharmacovigilance

## Abstract

**Background:**

Oxytocin and its analogue carbetocin are uterotonics whose prophylactic use is recommended to prevent postpartum haemorrhage, which is one of the leading causes of maternal deaths worldwide. However, both drugs can cause specific adverse effects and haemodynamic challenges.

**Aim:**

The aim of this work was to exploratively examine reports of adverse drug events of both drugs and to establish a comparative haemodynamic profile.

**Method:**

Using data extracted from the World Health Organization’s pharmacovigilance database VigiBase, a descriptive analysis was performed of all reports for oxytocin and carbetocin as a suspected or interacting drug followed by a disproportionality analysis for haemodynamic events. Reporting odds ratios (ROR) of carbetocin for hypertension, hypotension, tachycardia, and bradycardia were calculated, with oxytocin-related reports serving as comparators.

**Results:**

Oxytocin and carbetocin were mentioned as suspected or interacting drugs in 11,258 and 374 reports, respectively. Resulting RORs for carbetocin were 3.45 (95%CI: 1.72–6.92) for hypertension, 2.65 (1.64–4.28) for hypotension, 2.84 (1.79–4.49) for tachycardia, and 2.00 (0.87–4.60) for bradycardia, when compared to oxytocin. Of 231 patients for whom oxytocin-related tachycardia was reported, 2.6% died, and of 91 patients for whom bradycardia was reported, 2.2% died. No deaths were reported with carbetocin for any of the haemodynamic adverse events.

**Conclusion:**

Compared to oxytocin, carbetocin showed an elevated reporting for adverse hypertension, hypotension, and tachycardia in pharmacovigilance data. Clinicians should be aware of their patients' individual susceptibility and the possibility of haemodynamic deterioration until causal inferences are possible.

**Supplementary Information:**

The online version contains supplementary material available at 10.1007/s11096-023-01587-9.

## Impact statements

- Uterotonics are used in the management of postpartum haemorrhage, hence their haemodynamic effects must be considered.
- The structural differences of oxytocin and carbetocin have important consequences on their physiochemical and pharmacokinetic properties.
- Within the WHO database for individual case safety reports, there was a disproportionality in reporting for carbetocin, with higher odds of reporting for tachycardia, hypotension, and hypertension.

## Introduction

Uterine atony, which refers to decreased uterine muscle tone postpartum, is a leading cause of preventable maternal death. The World Health Organization (WHO) has recently updated its recommendations on the use of uterotonics to include their prophylactic use during the third stage of labour in all births, with the drug of choice being oxytocin [1]. Uterotonics are to be used in the management of postpartum haemorrhage, defined as blood loss of 500 mL or more after vaginal birth and 1000 mL or more after caesarean section birth within the 24 h period after childbirth [1].

Oxytocin is the synthetic drug of a neurohypophysial peptide hormone naturally produced in the hypothalamus [2], whilst its analogue carbetocin is an octapeptide with a modified disulphide bridge. The structural differences have important consequences on physiochemical and pharmacokinetic properties. First, oxytocin must be kept refrigerated, whereas carbetocin does not due to a heat-stable formulation. Second, oxytocin has a short plasma half-life of 1–6 min, whereas carbetocin has a plasma half-life of around 40 min [1]. When constant refrigeration and temperature-controlled transport are not available, oxytocin may, hence, present important quality reservations [3]. Although these quality reservations are debated [4], other uterotonics like carbetocin should be considered when concerns about the supply chain exist [1]. In addition, the longer half-life of carbetocin further results in the possibility of single dose administrations, whereas oxytocin must be given as an infusion [5].

Oxytocin and carbetocin both act on the oxytocin receptor, which is not only expressed in the uterus in the later stages of pregnancy, but is also present in the cardiovascular system and influences haemodynamic processes [2]. This consequently leads to adverse haemodynamic effects such as hypertension, hypotension, bradycardia, and tachycardia seen with both drugs [1, 5–7]. It can be argued that the specific haemodynamic profile of the analogue may differ due to their different half-lives. A 2018 Cochrane review that included a network analysis of uterotonics for the prevention of postpartum haemorrhage also reported differences in adverse effects [8]. In addition to nausea, vomiting, headache, fever, chills, abdominal pain, and diarrhoea, the only haemodynamic effect studied was hypertension. For hypertension, uncertainty was expressed for carbetocin because of the low level of certainty of evidence. Two clinical trials compared the haemodynamic profile of oxytocin and carbetocin directly [9, 10], but the conclusiveness of the results was unfortunately limited by small sample sizes. A recent consensus statement on uterotonics after caesarean section recommended the use of both oxytocin and carbetocin, as the differences between the two drugs are not yet clear [5].

### Aim

The safety and pharmacologic behaviour of uterotonics such as oxytocin and carbetocin are of great importance, as their ability to prevent maternal death also implies that they may be administered to vulnerable patients who are experiencing postpartum haemorrhage. The comparative haemodynamic safety profile of these analogues will help clinicians to make informed decisions when both drugs are available. The aim of this work was to exploratively examine reports of adverse drug events of both drugs and to establish a comparative haemodynamic profile.

### Ethics approval

This was an observational study with anonymous pharmacovigilance data.

## Method

### Database

Data were sourced from VigiBase via VigiLyze on October 20th, 2021. VigiBase is the WHO database for individual case safety reports (ICSRs) and it is the largest global database of spontaneous reports on adverse drug events [11]. Maintained by the Uppsala Monitoring Centre, VigiBase aggregates reports delivered from more than 170 member states of the WHO program for international drug monitoring and has been doing so since its establishment in 1968. ICSRs contain information on reported adverse reactions and events linked to drugs considered to be suspect, interacting, or concomitant. “Suspect” here means that the submitter suspected the drug as the main cause of the event, while “interacting” describes a drug-drug interaction and “concomitant” the mere presence of the drug in the medication list. More complete ICSRs also report on the established seriousness (i.e., whether medical intervention was required) and the outcome of the event. The reports also contain demographic information on the patients such as age, sex, weight, and height.

### Data analysis

The data were restricted to only include ICSRs for oxytocin and carbetocin, when reported to be the suspect or interacting drug. ICSRs were then grouped by descriptive population characteristics and summarised as counts with the corresponding percentages or as mean values with standard deviations (SD) for all reported adverse events. Missing values were excluded from descriptive statistics and reported separately.

Additionally, the four haemodynamic effects hypertension, hypotension, tachycardia, and bradycardia were expressed as corresponding Medical Dictionary for Regulatory Activities (MedDRA, [12]) preferred terms and identified in the individual ICSRs of oxytocin and carbetocin, respectively. The MedDRA terms are listed in Supplementary Table S1 (Online Resource 1). Reported age, use of additional adrenergics and uterotonics, and outcomes were explored separately for these haemodynamic events to investigate potential pharmacodynamic drug-drug interactions.

Reporting odds ratios (RORs) with corresponding 95% confidence intervals (CI) were calculated as measures of disproportionality [13]. RORs compare the number of reports mentioning the drug and adverse drug events in question with the reports for other drugs in the database [14]. For this analysis the ratio of the number of reports for the four haemodynamic effects to the number of reports for any other event with carbetocin formed the numerator, while the ratio of the number of reports for the four haemodynamic effects to the number of reports for any other event with oxytocin formed the denominator. This calculation allowed for a direct comparison between the two uterotonics, similar to the disproportionality analysis for haemorrhagic events during treatment with different non-vitamin K antagonist oral anticoagulants by Guo and colleagues [15]. When the 95%CI includes 1.00, no clear effect can be interpreted, and the result must be rejected as statistically not significant.

A sensitivity analysis was performed, aligning the available time periods for reports of the two drugs. This was undertaken to investigate to what extent the results were influenced by the longer availability of oxytocin.

All data handling and calculations were performed in RStudio, version 2022.02.3 build 492 [16], running R, version 4.2.0 [17], and the packages readxl [18], dplyr [19], and ggplot2 [20].

## Results

The source data included 11,276 ICSRs containing oxytocin and 377 ICSRs containing carbetocin. Of these, 11,258 ICSRs contained oxytocin and 374 ICSRs contained carbetocin as either suspect or interacting drug. The first entry for oxytocin was from 1969, the first one for carbetocin from 2002.

Table 1 shows the descriptive population characteristics for all reported adverse events in the database. The majority of cases related to oxytocin (74.9%) and carbetocin (83.2%) were attributed to women between 15 and 49 years. Around 60% of ICSRs originated from Asia, whilst Europe contributed 20.4% of oxytocin-related reports and 24.1% of carbetocin-related reports. ICSRs of carbetocin and oxytocin contained a similar number of reported additional drugs and uterotonics, with a mean of 0.9 (SD: 0.9) for oxytocin and 1.3 (2.5) for carbetocin. The most frequently reported concomitant drugs showed a high portion of overlap, with glucose, sodium chloride, and Ringer’s solution representing general infusions. Anaesthetics and analgesics were represented in the 10 most frequently reported concomitant drugs and included bupivacaine, fentanyl, paracetamol, propofol, and sufentanil. Other reported uterotonics included dinoprostone, misoprostol, and carboprost. In 0.3% of oxytocin-related ICSRs, carbetocin was reported as well, whilst oxytocin was reported in 16.0% of carbetocin-related ICSRs. Ephedrine as a vasoactive drug was reported in 0.6% and 4.0% of ICSRs for oxytocin and carbetocin, respectively. A higher percentage of cases was reported to be serious in carbetocin with 38.8% compared to 30.3% in oxytocin-related reports.

**Table 1:** Summary of all individual case safety reports (ICSRs) related to (suspect or interacting) oxytocin and carbetocin within VigiBase, the World Health Organization’s database for ICSRs

	Oxytocin (%)	Carbetocin (%)
Total ICSR	11,258 (100)	374 (100)
Total reactions	21,274 (100)	700 (100)
Patients		
Age [y]		
Mean (SD)	29.4 (8.4)	31.2 (7.5)
Under 1	177 (1.6)	5 (1.3)
1–14	24 (0.2)	1 (0.3)
15–24	1 774 (15.8)	29 (7.8)
25–34	4 933 (43.8)	185 (49.5)
35–44	1 622 (14.4)	96 (25.7)
45–54	258 (2.3)	2 (0.5)
Over 55	47 (0.4)	1 (0.3)
Not reported	2 433 (21.5)	55 (14.7)
Sex		
Female	10,402 (92.4)	368 (98.4)
Not reported	656 (5.8)	4 (1.1)
Reports		
Additional drugs		
Mean (SD)	0.9 (1.9)	1.3 (2.5)
Range	0–30	0–18
Additional uterotonics*		
Mean (SD)	0.1 (0.4)	0.2 (0.6)
Range	0–7	0–4
Most common concomitant drugs		
Glucose	945 (8.4)	9 (2.4)
Sodium chloride	941 (8.4)	23 (6.1)
Dinoprostone	524 (4.7)	1 (0.3)
Misoprostol	520 (4.6)	8 (2.1)
Bupivacaine	332 (2.9)	10 (2.7)
Fentanyl	287 (2.5)	16 (4.3)
Ringer’s solution	193 (1.7)	11 (2.9)
Cefazolin	161 (1.4)	19 (5.1)
Paracetamol	109 (1.0)	7 (1.9)
Propofol	105 (0.9)	2 (0.5)
Carboprost	76 (0.7)	20 (5.3)
Sufentanil	72 (0.6)	16 (4.3)
Ephedrine	71 (0.6)	15 (4.0)
Oxytocin	n.a	60 (16.0)
Carbetocin	31 (0.3)	n.a
Not coded	458 (4.1)	19 (5.1)
Origin of report		
Africa	159 (1.4)	14 (3.7)
America	1 446 (12.8)	31 (8.3)
Asia	6 929 (61.5)	219 (58.6)
Europe	2 292 (20.4)	90 (24.4)
Oceania	417 (3.7)	20 (5.3)
Not reported	15 (0.1)	0 (0.0)
Serious		
Yes	3 412 (30.3)	145 (38.8)
No	6 426 (57.1)	184 (49.2)
Not reported	1 420 (12.6)	45 (12.0)
Fatal		
Yes	164 (1.5)	3 (0.8)

*Uterotonics included: “Methylergometrine”, “Ergot alkaloids”, “Ergometrine”, “Dinoprostone”, “Gemeprost”, “Carboprost”, “Sulprostone”, “Misoprostol”

Table 2 shows the four haemodynamic effects hypertension, hypotension, tachycardia, and bradycardia for oxytocin and carbetocin, with counts and percentages of occurrences and reported seriousness. Tachycardia and hypotension were the most reported adverse reactions for both drugs. Within all investigated haemodynamic events, the reported seriousness was consistently higher for carbetocin-related ICSRs.

**Table 2:** Number of haemodynamic events of interest for oxytocin and carbetocin in VigiBase, the World Health Organization’s database for individual case safety reports

Outcome of interest	Oxytocin (N = 11,258)		Carbetocin (N = 374)	
	Occurrence (%)*	Serious (%)^†^	Occurrence (%)*	Serious (%)^†^
Hypotension	223 (2.0)	81 (36.3)	19 (5.1)	10 (52.6)
Hypertension	80 (0.7)	41 (51.2)	9 (2.4)	5 (55.6)
Bradycardia	91 (0.8)	47 (51.6)	6 (1.6)	4 (66.7)
Tachycardia	231 (2.1)	100 (43.3)	21 (5.6)	11 (52.4)

“Serious” equals whether medical intervention was required

*The percentage refers to all reports of the respective drug

^†^The percentage refers to the respective outcome and the respective drug

**Fig. 1:**
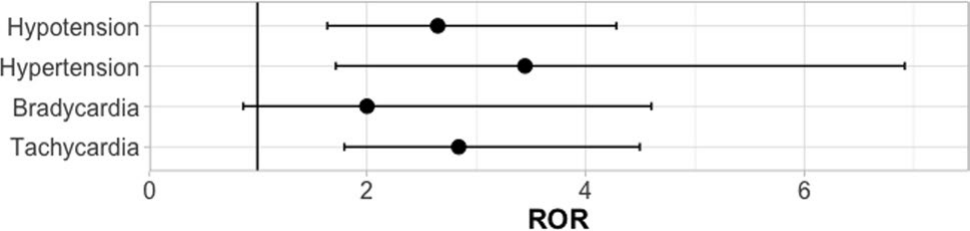
Reporting odds ratios with 95%-confidence intervals of adverse reactions of interest for carbetocin in relation to oxytocin in VigiBase, the WHO database for individual case safety reports

The RORs with corresponding 95%CIs for carbetocin in relation to oxytocin are displayed in Fig. 1. The ROR for hypertension was 3.45 (95%CI: 1.72–6.92) and 2.65 (1.64–4.28) for hypotension. For tachycardia, a ROR of 2.84 (1.79–4.49) was calculated, while that of bradycardia was determined to be 2.00 (0.87–4.60).

The sensitivity analysis, in which the time for reports of oxytocin was adjusted to the first available report of carbetocin (January 29th, 2002), limited the reports available for oxytocin from 11,258 to 10,960, with the number of haemodynamic events remaining the same. The resulting RORs for carbetocin were: 3.35 (1.67–6.73) for hypertension, 2.58 (1.59–4.17) for hypotension, 2.76 (1.75–4.37) for tachycardia, and 1.94 (0.85–4.48) for bradycardia.

Table 3 provides additional information on the reported outcomes, age, number of drugs, and number of uterotonics, stratified by the two drugs and the haemodynamic effects of interest. Among oxytocin reports for tachycardia, 2.6% reported death, and of oxytocin reports for bradycardia, 2.2% reported death. No deaths were reported with carbetocin. Full recovery was higher in the carbetocin group than in the oxytocin group after each type of haemodynamic event. In 4.2% of ICSRs for oxytocin reporting haemodynamic adverse events, the use of an adrenergic agent was reported. For carbetocin, adrenergics were reported in 9.1% of ICSRs reporting haemodynamic events. The use of an additional uterotonic was reported in 7.7% of ICSRs for oxytocin and in 10.9% of ICSRs for carbetocin.

**Table 3:** Age, number of drugs, number of uterotonics, and the reported outcomes, stratified by the two drugs and the haemodynamic effects of interest

	Hypotension		Hypertension		Tachycardia		Bradycardia	
	O	C	O	C	O	C	O	C
Total (%)^*^	223 (2.0)	19 (5.1)	80 (0.7)	9 (2.4)	231 (2.1)	21 (5.6)	91 (0.8)	6 (1.6)
*Outcome (%)*^*†*^								
Death	4 (1.8)	0	1 (1.3)	0	6 (2.6)	0	2 (2.2)	0
Not recovered/recovered with a sequela	2 (0.9)	1 (5.3)	5 (6.3)	0	2 (0.9)	1 (4.8)	3 (3.3)	0
Recovering	11 (4.9)	2 (10.5)	11 (13.8)	2 (22.2)	42 (18.2)	3 (14.3)	8 (8.8)	0
Fully recovered	150 (67.3)	14 (73.7)	47 (58.8)	7 (77.8)	143 (61.9)	11 (52.4)	59 (64.8)	5 (83.3)
Unknown	31 (13.9)	2 (10.5)	11 (13.8)	0	21 (9.1)	1 (4.8)	15 (16.5)	0
Not reported	25 (11.2)	0	5 (6.3)	0	17 (7.4)	5 (23.8)	4 (4.4)	1 (16.7)
Age [y], Mean (SD)	30.0 (6.2)	34.8 (14.8)	30.0 (6.8)	34.2 (5.8)	29.5 (8.2)	30.6 (3.6)	29.9 (8.6)	31.8 (3.3)
Other uterotonics present (%)^†^	12 (5.4)	2 (10.5)	18 (22.5)	2 (22.2)	15 (6.5)	2 (9.5)	3 (3.3)	0
Adrenergics present (%)^†^	12 (5.4)	2 (10.5)	5 (6.3)	0	9 (3.9)	2 (9.5)	0	1 (16.7)

O = Oxytocin; C = Carbetocin; Uterotonics included: “Methylergometrine”, “Ergot alkaloids”, “Ergometrine”, “Dinoprostone”, “Gemeprost”, “Carboprost”, “Sulprostone”, “Misoprostol”. Adrenergics included: “Etilefrine”, “Isoprenaline”, “Norepinephrine”, “Dopamine”, “Norfenefrine”, “Phenylephrine”, “Dobutamine”, “Oxedrine”, “Metaraminol”, “Methoxamine”, “Mephentermine”, “Dimetofrine”, “Prenalterol”, “Dopexamine”, ”Gepefrine”, “Ibopamine”, “Midodrine”, “Octopamine”, “Fenoldopam”, “Cafedrine”, “Arbutamine”, “Theodrenaline”, “Epinephrine”, “Amezinium Metilsulfate”, “Ephedrine”, “Droxidopa”

*The percentage refers to all reports of the respective drug

^†^The percentage refers to the respective outcome and the respective drug

## Discussion

Within the WHO database for individual case safety reports, there was a disproportionality in reporting for haemodynamic events when comparing carbetocin to oxytocin. Of the 374 ICSRs in which carbetocin was reported as a suspected or interacting drug, 55 contained haemodynamic events, compared with 640 of 11,258 ICSRs for oxytocin. In direct comparison, carbetocin showed an increased and significant ROR for tachycardia (2.84, 95%CI: 1.79–4.49), hypotension (2.65, 1.64–4.28), and hypertension (3.45, 1.72–6.92). The ROR for bradycardia was not statistically significant (2.00, 0.87–4.60).

The less favourable haemodynamic profile of carbetocin found here in pharmacovigilance data is not consistent with the existing literature. A Cochrane review from 2012 did not identify any significant differences in cardiovascular adverse drug reactions between carbetocin and oxytocin [21], but this finding was based on only one study with a total of 160 participants with vaginal delivery [22]. The Cochrane review from 2018 stated uncertainty whether carbetocin increases hypertension because of low levels of certainty of evidence. When compared to Syntometrine (oxytocin plus ergotamine), carbetocin showed a higher incidence of maternal tachycardia (risk ratio 1.68, 95%CI: 1.03–3.57) [23]. In a randomized control trial, Mannaerts et al. specifically investigated the adverse effects of carbetocin and oxytocin [10]. They found hypotensive effects for both drugs with a trend towards lower systolic blood pressure in the carbetocin group (14.4 ± 2.4 mmHg) than oxytocin (8.5 ± 1.8 mmHg). Unfortunately, the study did not achieve the calculated sample size to achieve the desired power of 80%. Moertl et al. [9] showed decreases in systolic blood pressure for both carbetocin and oxytocin, accompanied by increases in heart rate. The recovery was slower in the carbetocin group, which was attributed to the longer half-life of carbetocin. The differences between the drugs were deemed not to be significant, however, there were only 28 participants, limiting the study’s conclusiveness.

In clinical trials, drugs are usually studied in a small number of people, with restrictive inclusion and exclusion criteria [24]. The limitations of individual clinical trials also apply when the studies are later included in summative meta-analyses. It is only when medicines are used in large and diverse populations that rare adverse drug reactions can occur, which is one of the major strengths of pharmacovigilance analyses such as the one presented here. The Cochrane review from 2018 suggested that for maternal death no meaningful differences between all investigated uterotonics could be detected and that this outcome was rare across 59 included trials [8]. In these pharmacovigilance data, the reported seriousness of haemodynamic events was consistently higher for carbetocin-related ICSRs but compared to oxytocin their outcome was more frequently reported to be recovered or recovering. Notably, deaths were only reported for oxytocin-related haemodynamic events.

The 10 most common concomitant drugs for oxytocin and carbetocin were reported for both drugs. Major drug groups were analgesics, other uterotonics, and infusion solutions. Notably, 22.5% and 22.2% of ICSRs reporting on hypertension for oxytocin and carbetocin, respectively, reported the use of additional uterotonics. This may highlight increased hypertensive effects when two or more uterotonics must be used. The two Cochrane reviews from 2012 and 2018 concluded that the need for use of an additional uterotonic is slightly less for carbetocin than for oxytocin [8, 21]. Dinoprostone and misoprostol were more frequently reported with oxytocin than with carbetocin, whilst carboprost was more frequently reported with carbetocin in this dataset. Whilst the use of adrenergic agents was more frequently reported in haemodynamic adverse events for carbetocin, ranges from 0 to 16.7% do not account for the difference seen in the RORs. Although an underreporting of concomitant drugs must be expected, increased haemodynamic effects of carbetocin do not appear to be solely caused by pharmacodynamic drug-drug interactions.

The percentage of patients in the 35–44-year age group was higher for reports of carbetocin (25.7%) than oxytocin (14.4%), indicative of an older patient demographic for carbetocin. It is not possible to determine from the pharmacovigilance data the reason for this difference in the reporting profile. One speculative explanation is that carbetocin, as a newer agent, is still being used in high-income countries, where the percentage of parturients aged 35–44 years may be higher and pharmacovigilance reporting is also higher [25]. The likelihood of experiencing a haemodynamic deterioration is unlikely to be different for both drugs in the two main age groups of reports (25–34 years, 35–44 years).

In direct comparison to oxytocin, the ROR of carbetocin for bradycardia was not significant (2.00, 95%CI: 0.87–4.60). Arguably, bradycardia would mainly manifest as rebound bradycardia following adverse tachycardia. This was observed in the randomised trial by Moertl and colleagues, who described rebound bradycardia on average 200 s after tachycardia for oxytocin and 270 s after tachycardia for carbetocin [9]. In a pharmacovigilance report, it would then be up to the submitter to select the appropriate adverse effects to report, in which case only tachycardia could be selected and, hence, mask later occurring bradycardia. This may also explain why there were only six reports of bradycardia with carbetocin and highlights the limitations of pharmacovigilance data. Moertl and colleagues also found a non-significant trend towards less rebound bradycardia in patients receiving carbetocin [9], which would also be consistent with the results presented here.

### Limitations

Interpretations of pharmacovigilance analyses must be made with caution. Descriptive explorations and disproportionality calculations of spontaneous reports on adverse drug events are only capable of generating potential safety signals, and should not be misinterpreted as causal effects [26]. First, the number of reports within the database is directly influenced by the reporting behaviour of the submitters and, hence, biased by recent publications and discussions. This is especially true for newer drugs and treatment guideline changes, where healthcare professionals are urged to report their observations; an effect which may subside with time for established drugs and known adverse effects. Conversely, with time, more rare adverse drug events will be reported. These circumstances may distort the direct comparison between reports registered since 1969 and reports registered since 2002 as seen in this analysis. However, the sensitivity analysis, in which the time for reports of the two drugs was aligned, showed a negligible effect on the results. Differences in the number of reports were also found according to national income level, with high-income countries with well-established pharmacovigilance systems reporting the highest number of reports [25]. Although this again highlights the need for caution in interpreting pharmacovigilance analyses, the only significant difference was found for the therapeutic group of anti-infectives for systemic use. Second, the quality of ICSRs varies and contains measured and unmeasured levels of missingness and misclassification [27]. For an adverse event to be correctly entered, the submitters must choose the most appropriate term for the observation. This can be largely dependent on their knowledge and qualification. The submitters themselves are responsible for the quality of information of their reports, including their assessment of temporal relationships and assessments of causality. The quality assuring governance of national health authorities may be different across contributing countries and time.

## Conclusion

Within pharmacovigilance reports, carbetocin showed an elevated reporting for adverse hypertension, hypotension, and tachycardia compared to the current gold standard oxytocin. Clinicians should be aware of their patients' individual susceptibility and the possibility of haemodynamic deterioration until causal inferences are possible. This analysis adds to current knowledge of the haemodynamic effects of oxytocin and carbetocin and encourages a randomised controlled trial with sufficient power to confirm or refute these findings.

## Supplementary Information

Below is the link to the electronic supplementary material.

Supplementary file1 (PDF 63 KB)
