# Supplementary material for: An explorative analysis of pharmacovigilance data of oxytocin and its analogue carbetocin, with a focus on haemodynamic adverse effects
Source: Int J Clin Pharm. 2023 May 10;45(4):922–8. doi: 10.1007/s11096-023-01587-9 (PMC10366022; doi:10.1007/s11096-023-01587-9)
Supplement: Supplementary file 1 — Supplementary file1 (PDF 63 KB) [file 11096_2023_1587_MOESM1_ESM.pdf]

Supplementary Table 1 The four haemodynamic effects hypertension, hypotension, tachycardia, and bradycardia with their corresponding Medical Dictionary for Regulatory Activities (MedDRA®) preferred terms.

Outcome of Interest	MedDRA® terms
Hypotension	"Hypotension", "Orthostatic hypotension", "Diastolic hypotension", "Post procedural hypotension"
Hypertension	"Postoperative hypertension", "Maternal hypertension affecting foetus", "Accelerated hypertension", "Hypertensive crisis", "Malignant hypertension", "Malignant hypertensive heart disease", "Malignant renal hypertension", "Hypertensive emergency", "Hypertensive urgency", "Diastolic hypertension", "Essential hypertension", "Hypertension", "Secondary hypertension", "Systolic hypertension", "Labile hypertension", "Orthostatic hypertension", "Prehypertension"
Bradycardia	"Bradycardia", "Sinus bradycardia", "Central bradycardia"
Tachycardia	"Tachycardia", "Tachycardia paroxysmal", "Rebound tachycardia", "Atrial tachycardia", "Sinus tachycardia", "Supraventricular tachycardia", "Ventricular tachycardia", "Rebound tachycardia", "Junctional ectopic tachycardia"

Notes: The MedDRA® trademark is owned by the International Federation of Pharmaceutical Manufacturers and Associations on behalf of the International Council for Harmonisation of Technical Requirements for Pharmaceuticals for Human Use.

Article title: An Explorative Analysis of Pharmacovigilance Data of Oxytocin and its Analogue Carbetocin with a Focus on Haemodynamic Adverse Effects.

Journal name: International Journal of Clinical Pharmacy.

Author names: Dominik Stämpfli; Rebecca Dommrich; Sharon Orbach-Zinger; Andrea M Burden; Michael Heesen.

Affiliation and e-mail address of the corresponding author: Pharmacoepidemiology, Institute of Pharmaceutical Sciences, Department of Chemistry and Applied Biosciences, ETH Zurich, Switzerland; dominik.staempfli@pharma.ethz.ch.
